# A new insole measurement system to detect bending and torsional moments at the human foot during footwear condition: a technical report

**DOI:** 10.1186/s13047-015-0105-6

**Published:** 2015-09-09

**Authors:** Thomas Stief, Klaus Peikenkamp

**Affiliations:** Registered Association for the Encouragement of Research and Education Management in Orthopedic Footwear Technologies Germany, Ricklinger Stadtweg 92, D-301459 Hannover, Germany; Biomechanics Research Laboratory, Münster University of Applied Sciences, Bürgerkamp 3, D-48565 Steinfurt, Germany

**Keywords:** Foot, Forefoot, Human, Stress, Mechanical, Torque, Force, Torsional, Shoe, Orthopedics, Gait, Running

## Abstract

**Background:**

Stress occurring at the feet while wearing footwear is often determined using pressure measurement systems. However, other forms of stress, such as bending, torsional and shear loadings, cannot be detected in shoes during day-to-day activities. Nevertheless, the detection of these types of stresses would be helpful for understanding the mechanical aspects of various kinds of hard and soft tissue injuries. Therefore, we describe the development of a new measuring device that allows the reliable determination of bending and torsional load at the foot in shoes.

**Methods:**

The system consists of a measuring insole and an analogue device with Bluetooth interface. The specific shape of the insole base layer, the positions of the strain gauges, and the interconnections between them have all been selected in such a way so as to isolate bending and torsional moment detections in the medial and lateral metatarsal region. The system was calibrated using a classical two-point test procedure. A single case study was executed to evaluate the new device for practical use. This application consisted of one subject wearing neutral shoes walking on a treadmill.

**Results:**

The calibration results (coefficients of determination R^2^ > 0.999) show that bending and torsional load can be reliably detected using the measurement system presented. In the single case study, alternating bending and torsional load can be detected during walking, and the shape of the detected bending moments can be confirmed by the measurements of Arndt et al. (J Biomech 35:621–8, 2002).

**Conclusions:**

Despite some limitations, the presented device allows for the reliable determination of bending and torsional stresses at the foot in shoes.

## Background

Human feet fulfil several fundamental and complex tasks during daily activities. Among the most well-known are damping characteristics during the early stance phase of gait, flexibility for adaption to different surface conditions during barefoot walking and running, or rigidity for leverage during late stance phase of gait [1, 2]. During these activities, the soft and hard tissues of the feet are exposed to high mechanical loads, such as forces and moments [3–5]. Mechanical stress occurring at the feet can be detected using various kinds of plantar pressure measuring systems; for example, plates, insoles, or single sensors [6–8], force plates [2, 9], or calculated via inverse dynamics [10, 11] and other simulation techniques [12, 13]. Measuring plantar pressure is well established for detecting stress at the interface between foot and shoe [6, 14]. However, for various orthopaedic or systemic diseases, it can be of great interest to know about different forms of stresses at the human foot, such as shearing or bending stresses [5, 15]. Several studies mention shear forces as a major reason for ulceration in Diabetic Foot Syndrome (DFS) [16–18]. However, several problems occur with shear force sensors, which do not really allow their practical use (e.g. their size is insufficiently compact to easily use them in measuring insoles) [18, 19].

Bending is the most important load in mechanical engineering, material sciences, and biomechanics [20, 21]. The detection of bending stress via strain gauges has been well established for many years [22, 23]. Excessive bending stress is discussed in the aetiology of several pathologies and orthopaedic problems in the lower extremities, such as stress fractures in metatarsals [24, 25] and other bony conditions in the midfoot region; for example, extreme cases of DFS, and Sanders I- and II-type fractures in cases of neuropathic arthropathy (Charcot deformities) [26, 27]. In light of these issues, the detection of bending moments at the foot, especially at the metatarsal region, can be of great interest for understanding the mechanical aspects of those diseases. Furthermore, the detection of bending stress could be helpful in the verification of the efficacy of several nonsurgical orthopaedic treatments [28, 29]. In general, the moments acting on the metatarsals are detected by using inverse dynamic methods [10, 30, 31]. In contrast to the classical approaches, Arndt and colleagues measured bending strains at the second metatarsal of participants *in vivo* by an invasive method to understand more about bending loads at the midfoot and the causes of metatarsal stress fractures [32].

However, up to this point there is still no practical insole measurement system that allows for the determination of multidimensional stress [19], like bending or torsional moment detection. As a result, the development targets of the insole measurement system described above were:

(i) reliable bending and torsional moment detection with an insole in relevant regions of the human foot while wearing conventional footwear;
(ii) practical usage in orthopaedic practice (compact, low sensor positioning), and;
(iii) wireless data acquisition.

## Methods

This paper consists of two parts. Part I deals with the technical development details of the insole measurement system and Part II presents the practical use of the new system in a single case study. The participant provided written informed consent to participate in the single case and the study was in compliance with the Helsinki Declaration.

### The new insole system

**Fig. 1:**
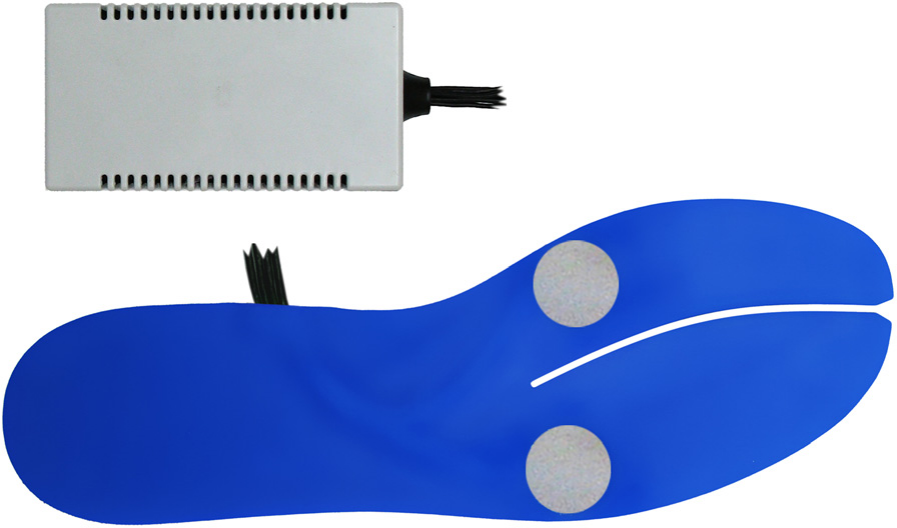
Insole measurement system configuration; top: analogue device with Bluetooth interface; bottom: Insole with measuring fields (grey spots)

A new, mobile, in-shoe measurement system was developed to detect bending and torsional moments in the human foot under conditions in which the subject was wearing footwear. The measurement system (Fig. 1) consists of: an insole with two measuring fields at the proximal forefoot region, and a data acquisition unit with Bluetooth connectivity and a battery power supply.

### Construction of the insole

**Fig. 2:**
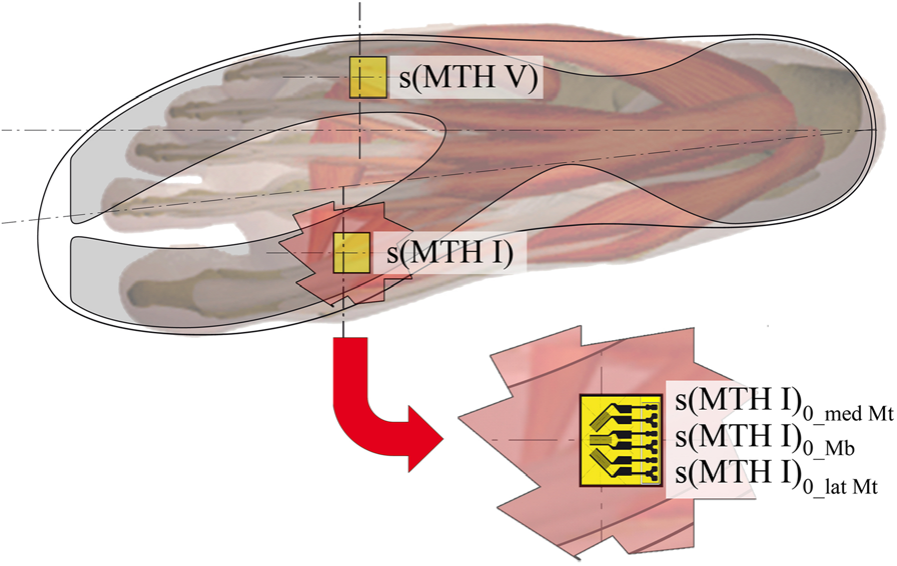
*top*: Insole with base layer shape (*grey translucent fork*) and sensor positions proximal metatarsal head one (MTH I) and five (MTH V) (*yellow rectangles*); *bottom right*: Sensor orientation with respect to anatomical structures (*red translucent cutout*)

The construction/design described follows classical principles of mechanical engineering and strain gauge measurement techniques [21, 23, 33, 34], which were transferred into a special, flexible measuring insole. 0°/45°/90° strain gauge rosettes (FAER-12B-35-S6EL; Vishay Measurements Group GmbH, Germany; Fig. 2, bottom right) were fixed mirror-inverted on both sides (top and down) of a specially shaped, thin layer of stainless steel (18Cr9Ni; height: 0.4 mm, E = 210,000 N/mm^2^, Rp_0.2_: 270 N/mm^2^; Record Metall Folien GmbH, Germany). The special shape of the stainless steel base was chosen to detect two independent bending and torsional “forefoot beams”, one at the medial and one at the lateral forefoot (Fig. 2, top: grey area). Two measurement fields with the inverted sensor array were installed on the base layer: one proximal to the first metatarsal head (MTH I), located on the medial forefoot beam; the second on the lateral forefoot beam proximal to the fifth metatarsal head (MTH V) (Fig. 2, top: yellow areas at the grey fork, medial: s(MTH I) and lateral: s(MTH V)).

We selected the positions of the measurement fields proximal to MTH I and MTH V for the following reasons:

(i) these regions experience high bending stress and cyclic loading at the human foot during daily living and sports activity [35, 36];
(ii) they represent locations of the main forefoot problems [37, 38] and;
(iii) they are locations of other hard tissue damage, e.g. Sanders I and II fractures related to Charcot deformities [26, 27].

**Fig. 3:**
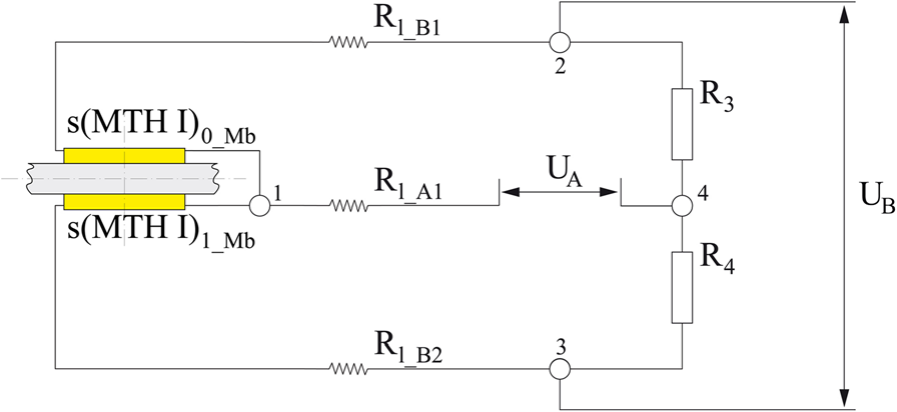
*left*: Schematic drawing of bending sensor array, sensors highlighted in yellow and base layer in grey; centre and *right*: Sensor integration into Wheatstone half bridge

The orientation of the strain gauge rosettes at each measurement field depends on the transverse flexion axis of the metatarsal joints. With regard to the 0°/45°/90° rosettes used in the test, this means that the centred 45° strain gauges were placed orthogonal to the joint axis (Fig. 2, bottom right). The strain gauge on the top side (s(MTH)_0_Mb_) was interconnected with the one on the bottom side of the measurement field (s(MTH)_1_Mb_) in adjacent branches of a half bridge circuit to create a bending transducer (Fig. 3). The sensor integrations at both measurement fields were chosen to detect positive voltage changes when the insole would be fixed proximal to the sensor arrays and the distal part of the forefoot beam would be bent upwards. This means that dorsiflexion moments (DFM) would result in positive voltage changes (Fig. 5, left). Plantarflexion moments (PFM) would be detected when the forefoot beam was flexed toward the bottom side of the insole.

**Fig. 4:**
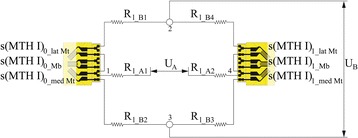
Torsion sensor integration into Wheatstone full bridge

**Fig. 5:**
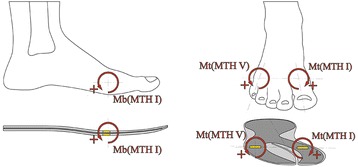
*left*: Sense of bending moment detection (curved arrows): positive bending direction represents positive voltage changes and dorsiflexion moments, whereas the negative direction describes negative voltage changes and plantarflexion moments; *right*: Sense of torsional moment detection (curved arrow): positive torsional direction represent positive voltage changes and external torsional moments, whereas the negative direction negative voltage changes and describes internal torsional moments

In order to build torque transducers, the 0° strain gauge (s(MTH)_0_lat Mt_) and 90° strain gauge (s(MTH I)_0_med Mt_) of the rosette on the top were connected into a full bridge circuit with the 0° strain gauge (s(MTH)_1_med Mt_) and the 90° strain gauge (s(MTH)_1_lat Mt_) of the rosette on the bottom side (Fig. 4). The interconnection described was selected for both sensor fields (one on the medial forefoot beam and one on the lateral beam) to detect external torsional moments (ETM) with respect to the longitudinal axis of the insole as positive voltage changes. This means that ETM should be detected if the cantilever beam is fixed proximal to the sensor array and the distal end is twisted around the longitudinal axis to its lateral side (Fig. 5, right). Negative values describe internal torsional moments (ITM).

High-viscosity silicon (TSE-397C; SH A15, E = 150 N/mm^2^; Momentive Performance Materials Inc., USA) was used to protect the sensor arrays and the cable circuits against destruction whilst the insoles were being used. In order to obtain a uniform surface and a typical medium width insole shape, the “bending fork” was covered on the top and bottom side with EVA (Lunalastik, 1.5 mm, SH A25, E = 250 N/mm^2^; nora systems, Germany) with cut-outs for the prepared measurement fields (Fig. 1, bottom: blue cover of the insole). The overall height of the completed insole was 3 mm.

### Setup of the measurement system

One important development objective was to ensure mobile and flexible usage of the measurement system in practice. Therefore, the torque and bending transducers of the insoles were connected by three-wire technique with shielded cables and a 4-channel bridge amplifier analogue device (GSV-4BT; bridge supply: 2.5 V, measurement range: 10 mv/V 16 bit, 125 Hz; ME-Messsysteme GmbH, Germany). Power was supplied by means of a lithium-ion rechargeable cell (CR18650-26 F; 2600 mAh, 0.2 C, 2.75 V discharge; Samsung SDI, Korea). For connectivity between the analogue device and a PC, a standard Bluetooth dongle with Bluetooth Standard 2.0 + EDR interface was used. The measurement setup is shown in Fig. 1. For data acquisition, recording, and processing, we used customised LabView (National Instruments, USA) based software.

### Calibrations

The project employed a classical, static, two-point cantilever bending and torsional test to calibrate bending and torsional moments. The bending calibration procedure for the first metatarsal region is described here as an example.

The medial forefoot beam of the insole under investigation was horizontally (independently from the lateral beam) clamped into place distal to the transducers. Strains over a 10-second period were detected while loading the cantilever forefoot beam consecutively with no weight and a set of calibration weights (0.005 kg, 0.02 kg, 0.05 kg, 0.1 kg, 0.2 kg, 0.5 kg, 1.0 kg, 2.0 kg; Accuracy class: M2; G&G GmbH, Germany) at the free, proximal end (distance between the centre of each mass and the centre of the strain gauge: 24 mm). This procedure was repeated five times for each condition. Between each measurement, the insole was released and fixed again as previously described. Afterwards, bending moments (Mb) were calculated for each measurement by using the linear bending theory equation, averaged for each weight condition, and finally the measured Mb were compared with the applied Mb. A similar procedure was performed to calibrate the measurement field proximal to MTH V.

For torsion calibrations, similar procedures as described above for bending calibrations were used. Only the positions of the weights were shifted 10 mm to the medial side of each cantilever beam with respect to the longitudinal axis of the insole.

### The pilot study using the new measurement system

Metatarsal bending and torsional moments at the right foot were detected during footwear conditions in a single case study by using the insole measurement system presented.

One male (27 years, 75 kg, 1.78 m, shoe size US 10) with no pathologies participated in the study. After inserting the insole into a neutral shoe (Samba; size US 10; adidas group, Germany), the participant put on the shoes, the analogue device was fixed with a belt at his waist, and the cable connections were secured by Velcro fasteners. Measurement was performed on a treadmill while the participant walked at his favoured speed (1.25 ± 0.03 m/s). Raw data were collected over a 60-second period per condition after the participant had a five-minute familiarisation phase. For data analysis, 40 consecutive step cycles were separated, normalised to 100 % of the gait cycle (accuracy: 101 data points), and averaged. To detect the initial contact of the foot, an algorithm was used that was known from EMG data preparations (Onset definition: triple standard deviation of the zero line) [39, 40].

## Results

### Calibrations

**Fig. 6:**
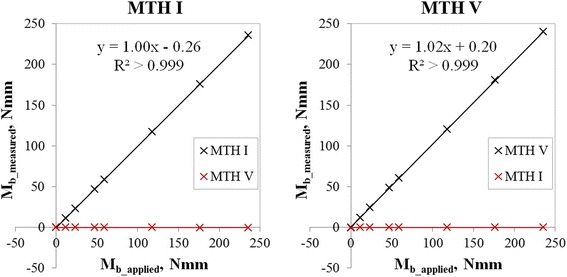
*left*: Bending moment (Mb) calibrations for MTH I sensor array showing linearity (black curve) and cross talk effects < 0.5 % (red curve); *right*: Mb calibrations for MTH V sensor array showing linearity (black curve) and cross talk effects < 0.5 % (red curve)

**Fig. 7:**
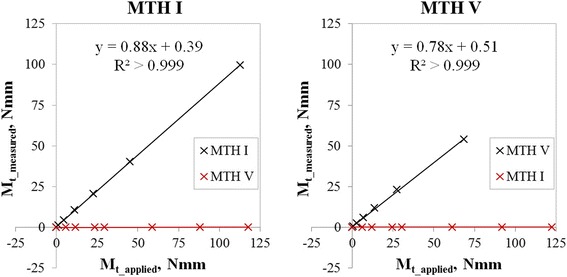
*left*: Torsional moment (Mt) calibrations for the MTH I sensor array showing linearity (black curve) and cross talk effects < 0.5 % at unloaded channels (red curve); *right*: Mt calibrations for the MTH V sensor array showing linearity (black curve) and cross talk effects < 0.5 % (red curve)

The coefficients of variation (R^2^) for the bending moment calibrations of both measurement fields were greater than 0.999 and the linearity factors were close to 1.0 (1.00 for MTH I sensor array and 1.02 for MTH V sensor array) (Fig. 6). Consequently, linear regression equations are sufficient to determine the calibration factors. Crosstalk effects were found with less than 0.5 % of the unloaded sensor arrays (Fig. 6: red crosses and red lines). R^2^ for the torsional moment calibrations of both measurement fields were greater than 0.999 as well, and the linearity factors were 0.88 at the measurement field proximal MTH I and 0.78 proximal MTH V (Fig. 7). Again, crosstalk effects amounted to less than 0.5 % of the unloaded sensors (Fig. 7: red crosses and red lines).

### The pilot study using the new measurement system

**Fig. 8:**
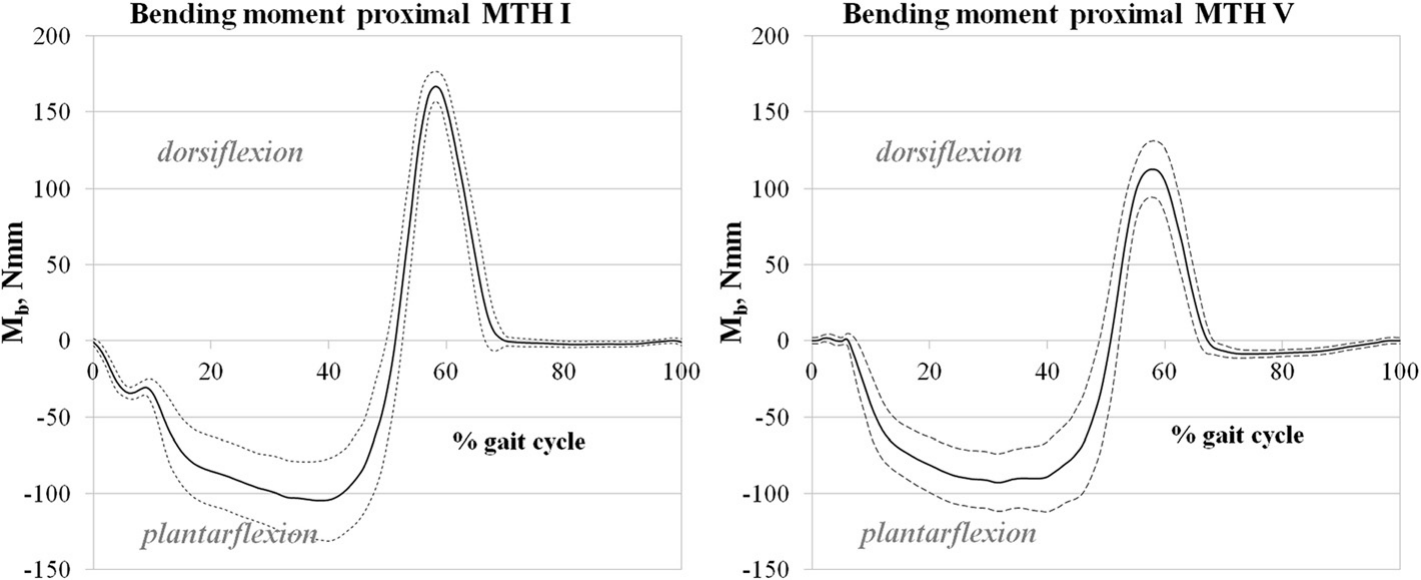
*left*: Bending moment (Mb) proximal MTH I during gait cycle: positive values represent dorsiflexion, negative values plantarflexion moments; *right*: Mb during gait proximal MTH V

Figure 8 (left) shows the averaged bending moments detected using the proximal MTH I sensor array. Positive values represent dorsiflexion moments (DFM) and negative values represent plantarflexion moments (PFM). From initial contact to 51 % of the gait cycle, a PFM with a maximum of 105 ± 26 Nmm at 39 % gait cycle was detected. At 52 % of the gait cycle, the bending changes into dorsiflexion tension with its maximum at 58 % of the gait cycle with 167 ± 10 Nmm. After the maximum, dorsiflexion stress decreases and crosses the zero line at 70 % gait cycle and reaches a PFM at ≈ 2 ± 1 Nmm at 75 % gait cycle. This level does not really change until the end of the gait cycle.

The curve of the bending moment (normalised to 100 % of the gait cycle) seen via the proximal MTH V sensor shows a similar shape as the one of proximal MTH I (Fig. 8, right). Only at the beginning of the gait cycle (0-7 %) does a low DFM (1 ± 1 Nmm) occur at MTH V. After that, PFM rises, with its maximum (93 ± 20 Nmm) at 32 % of the gait cycle. At 51 % of the gait cycle, bending changes into DFM. The DFM maximum can be detected at 58 % of the gait cycle, with 113 ± 18 Nmm. DFM rapidly decreases thereafter, crosses zero (at 71 % of the gait cycle) and levels out until the end of gait by PFM: 5 ± 3 Nmm.

**Fig. 9:**
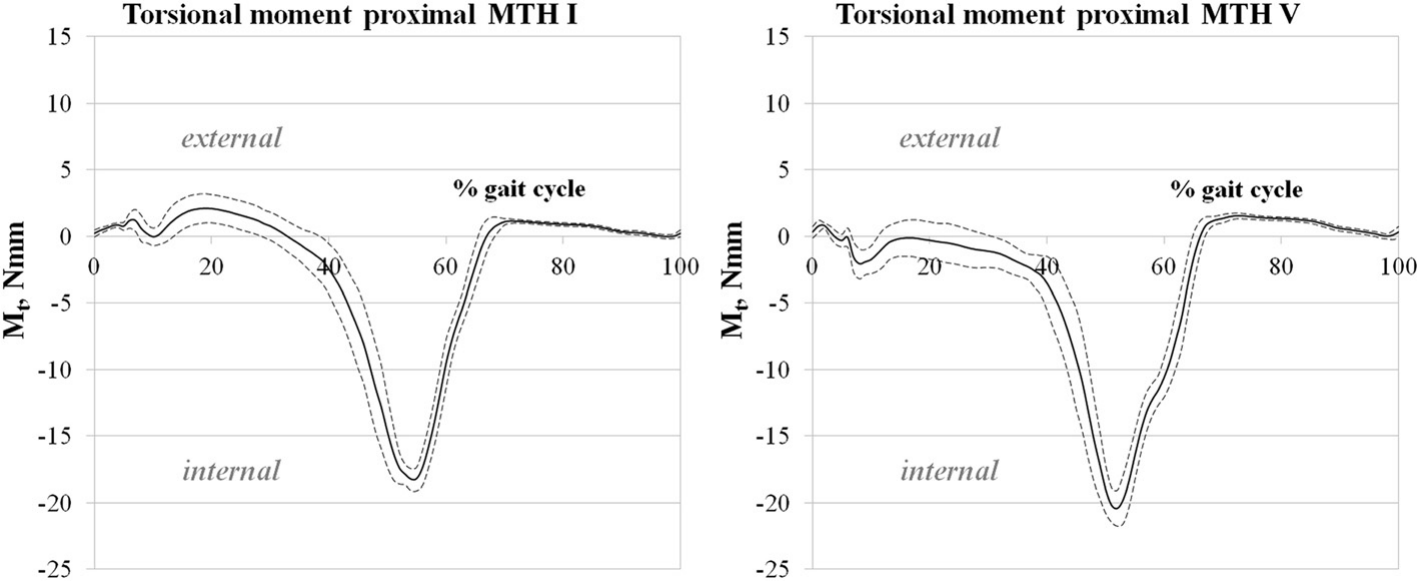
*left*: Torsional moment (Mt) proximal MTH I during gait cycle; positive values represent external torsional (ETM), negative values internal torsional moments (ITM); *right*: Mt proximal MTH V during gait cycle

The torsional stresses detected using sensors MTH I and MTH V are plotted in Fig. 9. As previously described, positive values indicate internal torsional moments (ITM), and negative values represent external torsional moments (ETM). From 0 % of the gait cycle to 32 % of the gait cycle, ETM (maximum: 2 ± 1 Nmm, 19 % gait cycle) can be seen in readings from the proximal MTH I sensor (Fig. 9, left). At 33 % of the gait cycle, torsional load changes into ITM, with a maximum (18 ± 1 Nmm) at 54 % of the gait cycle. After that, ITM decreases, crosses zero at 71 % of the gait cycle and levels out (ETM: 1 ± 0 Nmm) until the end of the gait cycle.

With respect to the MTH V sensor, ETM occurs during the early stance phase until 4 % of the gait cycle. After that, ITM can be detected, but it decreases immediately and reaches a neutral level (0 ± 1 Nmm) at 17 % of the gait cycle again, followed by another ITM increase with a maximum of 20 ± 1 Nmm, at 55 % of the gait cycle. Subsequently, after the maximum, the ITM drops and changes into ETM at 67 % of the gait cycle. During swing phase, ETM (maximum: 2 ± 0 Nmm) with an average level of 1 ± 0 Nmm can be detected (Fig. 9, right).

## Discussion

R^2^ was found to be greater than 0.999 for both bending and torsional moments at MTH I and MTH V. These results show that the measuring system presented above allows for the reliable and reproducible measurement of bending and torsional loads using a flexible insole. The linearity factors for the bending moments with both sensor arrays were nearly 1.0 and for the torsional moments ≈ 0.9 at MTH I and ≈ 0.8 at MTH V. This means that the entire insole setup with silicon and EVA protection, cable connections, etc. has a negligible influence on bending characteristics of the forefoot beams, though it could have an influence on torsion. The low crosstalk effects show that this insole system allows for independent measurements of both bending and torsional moments at the medial and lateral forefoot beam of the insole.

**Fig. 10:**
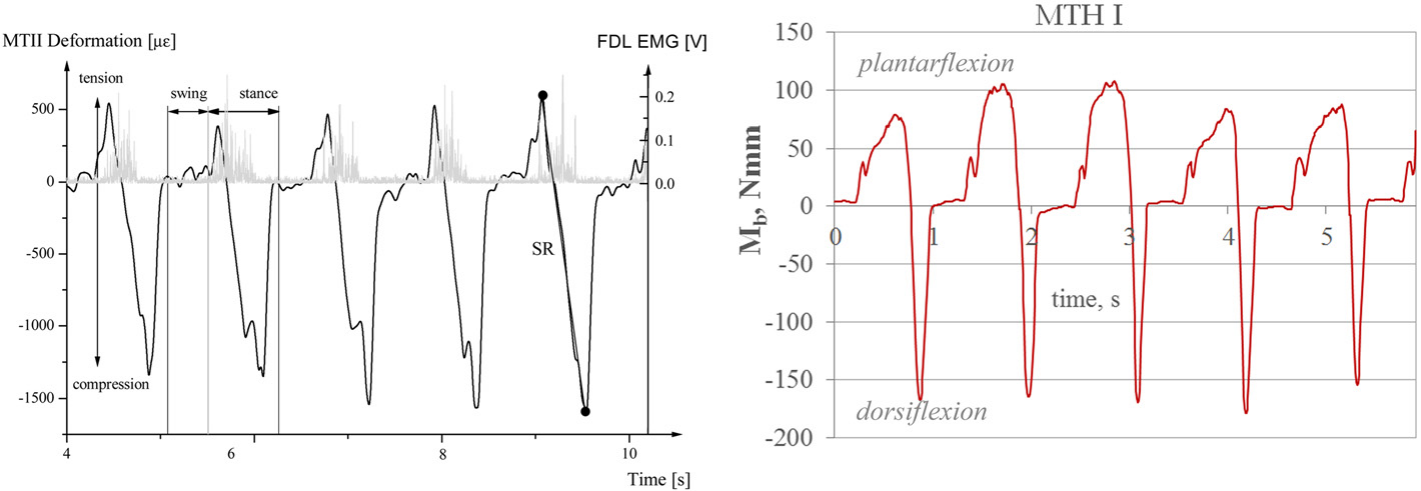
*left*: Data collected by Arndt et al. [32], black line: strains of the second dorsal metatarsal during walking (≈ five gait cycles), *grey*: M. flexor digitorum longus (FDL) activity, “Reprinted from Journal of Biomechanics, 35, Arndt et al. Effects of fatigue and load variation on metatarsal deformation measured *in vivo* during barefoot walking, 621–628, Copyright 2002, with permission from Elsevier”; *right*: Mb detected at the sensors proximal MTH I showing similar time characteristics; *both*: negative values represent dorsiflexion, positive values plantarflexion strain/stress

Under typical conditions, in which the participant wore footwear, we detected bending and torsional moments during treadmill walking with respect to the shoe condition. The time-variable characteristics of bending load on the MTH I and MTH V sensors are comparable to results published by Arndt et al. in 2002 (Fig. 10). They measured dorsal strain *in vivo* using an instrumented staple at the second metatarsal. They found tensional strain during the first part of the gait cycle, which changed into compression stress with its maximum at ≈ 65 % of the gait cycle. During swing phase of gait, nearly no stresses were found in this study. Due to their measuring configuration, any detected tensional stress could be interpreted as plantarflexion stress and any compression stress was seen as dorsiflexion stress. Our insole system also was able to detect plantarflexion stress (PFM, from 0 % of the gait cycle until ≈ 50 % of the gait cycle), followed by dorsiflexion stress (DEM, with a peak at 68 % at the MTH V and at 70 % at the MTH I sensors). Additionally, during the swing phase, no bending moments could be ascertained.

The major difference between the stress at the forefoot, which Arndt and colleagues [32] found, and the result presented are that they detected *in vivo* bending strains directly at the dorsal bone and we detect bending stress at the interface of the foot surface and shoe. For two reasons, it is not possible to directly compare the absolute values of the results of both measurement techniques. One reason for this is the complex and heterogeneous structure of the various soft and hard tissues of the foot that exist between the plantar foot surface and the dorsal side of the metatarsals. The second reason is due to the measurement technique that Arndt and colleagues used. The setup used in our insole, while facilitating the detection of metatarsal strain, cannot determine the absolute values of bending moments [22, 32, 36]. Nevertheless, even though the relationship between bending strain, tension, and bending loads is very complex, the characteristics and the shape of both detected parameters are quite comparable [21, 23, 34]. One remarkable finding in the single case study, which supports this conclusion, is the finding of alternating bending stress (PFM and DEM), as Arndt et al. also noted [32]. In general, inverse dynamic approaches do not show this characteristic of MTH bending loads [10, 30, 41]. It can also not be detected by plantar pressure measurement techniques. The alternating bending phenomena could be an objective of further studies, because we found relatively high plantarflexion stress compared to the dorsiflexion stress (MTH V; DEM: 113 Nmm, PFM: 93 Nmm), which is in contrast to *in vitro* strain gauge measurement studies [24, 36]. The torsional moments detected were quite low compared to the bending moments. ITM generally occurs at both sensor arrays with the maxima at almost 53 % of the gait cycle, which corresponds to the terminal stance phase. However, the graphs of the torsional loads are quiet similar to shear-force measurement results [16]. One explanation for the internal torsional stress on both forefoot beams may be the progress of the centre of pressure during the late stance phase of the gait [42]. The observed moments should not be confused with internal moments. With the measuring system presented in this article, moments at the interface between the foot and shoe, which are responsible or otherwise involved in the stresses, are measured. This is also a reason why absolute values were not discussed in this article, because further data, such as normative data under several conditions (e.g. barefoot) still have to be obtained to get a comparable data set. Nevertheless, it is possible to conclude that the following goals can be successfully demonstrated:

(i) reliable bending and torsional moment detection with an insole in relevant regions of the human foot while wearing conventional footwear;
(ii) practical usage in orthopaedic practice (compact, low sensor positioning), and;
(iii) wireless data acquisition.

There are four limitations of the existing measurement system and the single case. Firstly, the major limitation of the insole is the small number of sensor fields. However, it is easily possible to increase the volume of sensors on the insole and to modify the shape of the insole to get several fore, mid, or rearfoot beams. Secondly, a further limitation of the system is the 3 mm height of the insole. Thirdly, the relatively low measurement frequency (125 Hz) allows stress detection during static and everyday tasks, but not during sports activities. Finally, during the single case, only bending and torsional load of the right foot were detected. However, for height compensation, a similar insole without sensors was used for the left foot. The aforementioned limitations/problems can also be optimised in a further stage of expansion of the system without any problems as well. We simply wanted to verify whether it was in principle possible to produce measurements of bending and torsional moments under conditions in which a participant wears footwear with the insole system described above.

## Conclusion

The calibration results and the results of the single case study show that the new insole measurement system presented allows for mobile, reliable, and easy detection of bending and torsional moments at the forefoot. The easy bending and torsional stress detection at the foot may be of interest for both daily practice and/or research. Further research is needed before the results of the single case can be generalised.

## Abbreviations

DFM: Dorsiflexion moment
DFS: Diabetic Foot Syndrome
ETM: External torsion moments
ITM: Internal torsion moments
Mb: Bending moment
Mt: Torsional moment
MTH I: First metatarsal head
MTH V: Fifth metatarsal head
PFM: Plantarflexion moment
